# Correction: Epigenetic Silencing of Peroxisome Proliferator-Activated Receptor γ Is a Biomarker for Colorectal Cancer Progression and Adverse Patients' Outcome

**DOI:** 10.1371/annotation/07419a37-33f1-4e4a-adc9-b186f305bae5

**Published:** 2011-01-11

**Authors:** Massimo Pancione, Lina Sabatino, Alessandra Fucci, Vincenzo Carafa, Angela Nebbioso, Nicola Forte, Antonio Febbraro, Domenico Parente, Concetta Ambrosino, Nicola Normanno, Lucia Altucci, Vittorio Colantuoni

A grant number was missing from the original funding section. The authors wish to add the following: The EU funding refers to the EU 7th frame program 'CancerDip' project contract n° 200620 to L.A.

